# Dietary Sugars and Endogenous Formation of Advanced Glycation Endproducts: Emerging Mechanisms of Disease

**DOI:** 10.3390/nu9040385

**Published:** 2017-04-14

**Authors:** Manuela Aragno, Raffaella Mastrocola

**Affiliations:** Department of Clinical and Biological Sciences, University of Turin, Corso Raffaello 30, 10125 Turin, Italy; manuela.aragno@unito.it

**Keywords:** advanced glycation end products, fructose, glucose, lipogenesis, sphingolipids, NLRP3, Nrf2, mitochondrial dysfunction, oxidative stress

## Abstract

The rapid increase in metabolic diseases, which occurred in the last three decades in both industrialized and developing countries, has been related to the rise in sugar-added foods and sweetened beverages consumption. An emerging topic in the pathogenesis of metabolic diseases related to modern nutrition is the role of Advanced Glycation Endproducts (AGEs). AGEs can be ingested with high temperature processed foods, but also endogenously formed as a consequence of a high dietary sugar intake. Animal models of high sugar consumption, in particular fructose, have reported AGE accumulation in different tissues in association with peripheral insulin resistance and lipid metabolism alterations. The in vitro observation that fructose is one of the most rapid and effective glycating agents when compared to other sugars has prompted the investigation of the in vivo fructose-induced glycation. In particular, the widespread employment of fructose as sweetener has been ascribed by many experimental and observational studies for the enhancement of lipogenesis and intracellular lipid deposition. Indeed, diet-derived AGEs have been demonstrated to interfere with many cell functions such as lipid synthesis, inflammation, antioxidant defences, and mitochondrial metabolism. Moreover, emerging evidence also in humans suggest that this impact of dietary AGEs on different signalling pathways can contribute to the onset of organ damage in liver, skeletal and cardiac muscle, and the brain, affecting not only metabolic control, but global health. Indeed, the most recent reports on the effects of high sugar consumption and diet-derived AGEs on human health reviewed here suggest the need to limit the dietary sources of AGEs, including added sugars, to prevent the development of metabolic diseases and related comorbidities.

## 1. Dietary Sugars as a Risk for Health

### 1.1. Sugar Consumption in Modern Society

Significant modifications of human diet composition, as well as of frequency and timing of energy and nutrients intake, have been observed in the last forty years, representing potential risk factors for the development of metabolic diseases. An increase of the daily energy intake of 505 kcal, corresponding to 25%, from 1970 to 2010 [1] has been described, and a rise in *per capita* food consumption from 5 kg to 70 kg per year from 1800 to 2006 has been estimated [2]. Based on these observations, the current Guidelines of Nutrition and Health Recommendations suggest that a healthy diet must provide no more than 5% of total energy intake as simple sugars. In contrast, currently, 13% of the American population consumes over 25% of their daily energy intake as sugar [3].

In addition, clinical evidence suggests that sugar-sweetened foods create psychological dependence [4]. Indeed, clinical observations report that removing sugar from the diet causes effects like hyperactivity, conduct problems, and mental disturbances [5,6].

On the other hand, results from experimental models confirm that the consumption of sugar-added foods is associated with increased risk for obesity [7], as well as cardiovascular diseases [8,9], metabolic disorders [10], non-alcoholic fatty liver disease (NAFLD) [11,12], and cognitive decline [13]. Actually, some controversial conflicts over the role of an excessive intake of sweetened foods and beverages on public health and the interest of food and beverage industry have been debated [14]. Recent reviews report several critical issues on the criteria and low quality evidence used for recommendations and guidelines [15,16,17].

However, sugar added to foods and drinks adds considerable calories without any benefits and may take the place of other nutrient-dense foods in the diet. Thus, many of the clinical and epidemiological observations indicating that excess glucose and fructose intake exacerbates metabolic complications in different tissues are possibly due to the increased calories intake. At present, there seems to be reliable evidence of results obtained from experimental models about the negative effects of high dietary sugar intake, but no clear reliable evidence indicating daily caloric thresholds for sugar intake to exert negative health effects in human.

### 1.2. Fructose Consumption and Prevalence of Metabolic Diseases

Sucrose, formed by 50% fructose and 50% glucose joint by a glycosidic bond, has been the most easily consumed sweetener in the last decade. The introduction of corn-derived sweeteners, in particular of high-fructose corn syrup (HFCS), which is provided with high sweetening power, organoleptic properties, the ability to confer a long shelf life and to maintain a long-lasting hydration in industrial bakeries, together with its low cost, has rapidly reduced the use of sucrose in many industrial preparations [2]. The fructose content in HFCS is in a range between 42% and 55% of total sugar, and both fructose and glucose are in their pure form, without glycosidic bond. The commercial use of HFCS as a common sweetener has strongly raised the content of fructose in the human diet through consumption of sweetened beverages, tea, coffee, sodas, snacks, and bakeries.

Some epidemiological studies show an association between fructose-containing sweeteners intake and body weight gain [8,18]. Moreover, clinical evidence indicates that a high-fructose diet is associated with the onset of dyslipidemia, insulin resistance, and related metabolic diseases [19,20]. These observations in humans have been confirmed and further extended by animal studies indicating that fructose added to the diet contributes to the development of obesity, inflammation, and decrease of the activity of the mitochondrial metabolism regulator peroxisome proliferator-activated receptor alpha (PGC1-alpha) [10,21]. High-sugar fed animals are commonly used as suitable experimental models to highlight pathogenic mechanisms related to metabolic diseases onset following imbalanced high-calories diets [22,23,24]. However, although the negative effects of fructose have been observed and described in these models, the mechanisms proposed are not yet exhaustive to define whether dietary fructose, when consumed in moderate amounts, is actually deleterious for human health.

### 1.3. Dietary Fructose and Glucose Metabolism

**Scheme 1:**
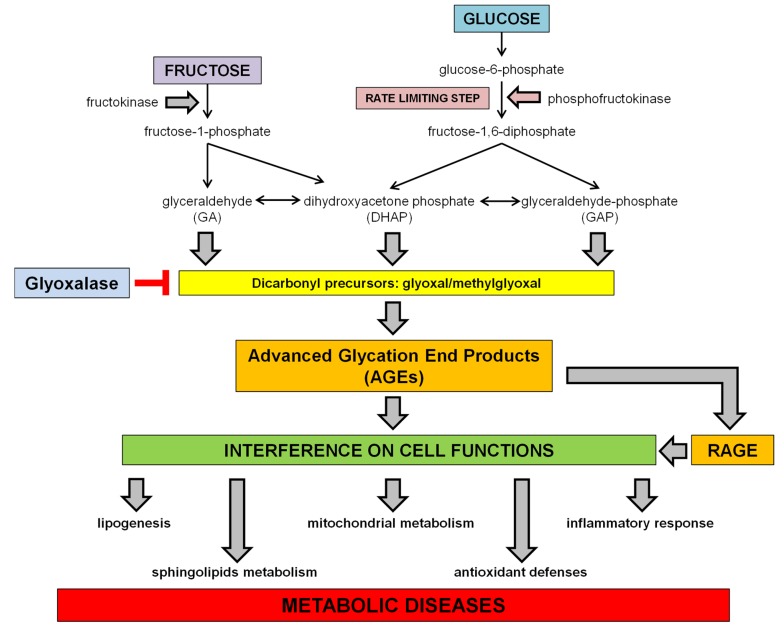
**Fructose and glucose metabolism and proposed effects for dietary sugar-induced glycation.** Fructose bypasses the rate limiting step of glycolysis leading to a faster generation of dicarbonyl precursors than glucose. AGE accumulation in tissues can interfere with many protein functions contributing to the onset of metabolic diseases and related comorbidities.

Dietary sugars, including glucose and fructose, are absorbed in the small intestine, but the absorptive capacity for fructose is lower than for glucose or sucrose. However, the addition of glucose, as in case of HFCS-added foods, facilitates the absorption of fructose [25]. After absorption, nevertheless, the metabolism of the two monosaccharides follows different pathways, since glucose can be used directly by the cells to produce energy in a variety of organs, while fructose is primarily metabolized in the liver, which takes up at least 50% of the initial fructose flux [26]. In the cells, glucose is phosphorylated by hexokinase to glucose-6-phosphate, which is then converted to fructose-1-6-diphosphate by the phosphofructokinase, the rate limiting enzyme of glycolysis. The cleavage of fructose-1,6-diphosphate by fructose diphosphate aldolase produces the triose phosphate intermediates dihydroxyacetone phosphate (DHAP) and glyceraldehyde-3-phosphate (GAP). Conversely, the fructose metabolism bypasses the need of insulin and the phosphofructokinase regulation step, and enters glycolysis directly at the step of triose phosphate intermediates generation. Indeed, fructose is phosphorylated by ATP to fructose-1-phosphate, catalyzed by fructokinase. Fructose-1-phosphate is then split by hepatic aldolase B into glyceraldehyde (GA) and DHAP, which can be both converted to GAP. These metabolites are at the centre of metabolic crossroads that lead to glycolysis, gluconeogenesis, glycogenesis and lipogenesis [27] (see Scheme 1).

## 2. Dietary Sugars and Glycation

### 2.1. Advanced Glycation Endproducts (AGEs)

AGEs are toxic compounds deriving from non-enzymatic glycoxidation reactions of reducing sugars with proteins, which then result as being structurally and functionally compromised [28]. Protein glycation is initiated by a nucleophilic addition reaction between the free amino group from a protein, lipid or nucleic acid and the carbonyl group of monosaccharides. This reaction forms a reversible Schiff base, which rearranges over a period of days to produce ketoamine or Amadori products. The Amadori products undergo dehydration and rearrangements followed by other reactions involving dicarbonyl compounds, such as cyclization, oxidation and dehydration, to form irreversible AGEs [29]. Proteins glycation occurs in vivo in physiological conditions and the Maillard reaction represents a type of post-translational modification of molecules that takes place slowly and continuously throughout the life span, driving AGE accumulation in tissues during ageing [20]. For this reason, AGEs have been involved in the pathogenesis of age-related diseases, such as neurodegenerative diseases, atherosclerosis, and chronic inflammatory diseases [30], but in particular conditions, such as diabetes and insulin resistance, the accumulation of AGEs is accelerated, leading to early developing of comorbidities [31]. Indeed, hyperglycemia is known to induce high rates of protein glycation, which is responsible for the development of long-term complications [30].

### 2.2. In Vitro Protein Glycation of Different Sugars

It has been a long time since studies started to investigate the glycative potential of different monosaccharides by in vitro incubations with physiologically relevant proteins such as haemoglobin and serum albumin [32,33]. The first comparison among sugars for their non-enzymatic reactivity with haemoglobin has been published by Bunn and Higgins in 1981, showing that fructose has a reaction rate 7.5-fold higher than glucose [32]. A few years later, Suarez et al. found that the rate of glycoxidation of bovine serum albumin by fructose was about 10 times higher than that by glucose [34]. Since then, other studies have confirmed the sequence ribose > fructose > glucose for the rate of glycoxidation [35,36], even if some of them failed to detect relevant differences on the glycation potential of fructose compared to glucose in terms of time and intensity of browning during incubation with amino acids [37,38].

**Scheme 2:**
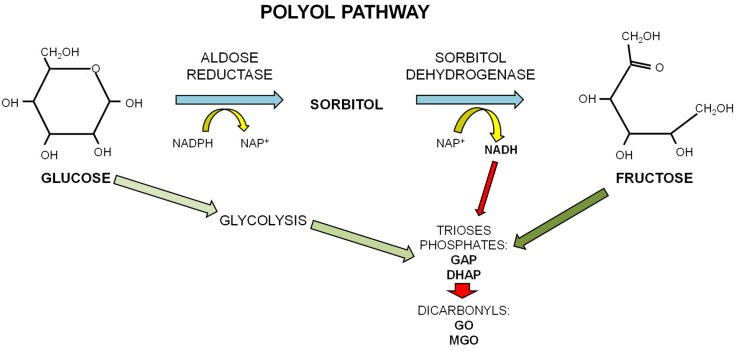
**The polyol pathway.** In conditions of excess of glucose, as occurring in diabetes, glucose undertakes the polyol pathway to be converted to fructose through the consecutive action of aldose reductase and sorbitol dehydrogenase. This alternative pathway for the metabolism of glucose leads to the increase in fructose levels in tissues of diabetic patients and of the NADH/NAD+ ratio that contrasts GAP-dehydrogenase activity leading to accumulation of the triose phosphates GAP and DHAP, precursors of the dicarbonyls compounds glyoxal (GO) and methylglyoxal (MGO).

The papers reporting a different kinetic in AGE formation from glucose and fructose have attributed it to the different forms in which the two sugars exist in physiological conditions. Glucose, a d-aldohexose, exists in solution as a stable ring structure. Since only the open chain form of sugar can react with an amino group in protein to form a Schiff base, the high reactivity of fructose may reflect its higher quote of existing as an open chain in solution than glucose [32,33]. The main glycating sugar present in the body at the highest concentration is glucose. However, fructose can be produced in conditions of hyperglycemia by the polyol pathway where glucose is converted to fructose through the consecutive action of aldose reductase and sorbitol dehydrogenase. Oxidation of sorbitol by sorbitol dehydrogenase (SDH) yields NADH and causes an increase in the ratio NADH/NAD+ that may contrast GAP-dehydrogenase activity, thus leading to accumulation of the triose phosphates (see Scheme 2). This conversion of excessive glucose to fructose leads to an increase in fructose levels in tissues of diabetic patients [39]. A second aspect linking high fructose levels to AGE production is related to the peculiar fructose metabolism that evokes rapid generation and accumulation of GAP and DHAP, both effective glycating agents and precursors of the dicarbonyls compounds glyoxal and methylglyoxal, which, in turn, are precursors of more stable AGEs [40].

There are differences between glucose and fructose also concerning the glycating process, since the rearrangement of the Schiff base derived from fructose generates Heyns products that are quite different from the Amadori products formed by glucose, for they undergo a more rapid conversion to irreversible AGEs [34]. However, in addition, the opposite observation of a slower rate of Maillard fluorescence generation for the Haynes products derived from fructose compared to Amadori products derived from glucose has been reported [41].

### 2.3. Dietary Intake of Exogenous AGEs and of Simple Sugars as Potential Sources for Endogenous AGEs

In recent years, several studies have highlighted some dietary aspects that can influence extra- and intra-cellular accumulation of AGEs.

First, the high-temperature and long-time cooking of foods can generate AGEs that are exogenously introduced with the diet [42]. A relevant number of intervention studies, reviewed by Kellow and Savige, have investigated the effects of an AGE-restricted diet on inflammatory markers and insulin sensitivity. Their meta-analysis of the literature provided evidence that, although the reduction of AGEs introduced with foods is associated to reduced CML plasma levels, a direct relationship with improved insulin sensitivity and inflammatory profile is still not clearly demonstrated by high quality clinical trials [43].

Second, emerging evidence indicates that high dietary simple sugars consumption can represent a substantial source of endogenous AGEs [44]. In light of the in vitro observation that fructose is much more reactive than glucose in generating glycation precursors, the modern nutrition implicating a much higher fructose intake than decades ago is an important factor contributing to the increase in plasma fructose concentration in healthy subjects [45], possibly contributing to AGE formation. Thus, among the sugars mostly used for sweetening of foods and drinks, the fructose might represent the most hazardous for AGE accumulation. Population studies have evidenced that, in individuals with NAFLD, the concomitant presence of metabolic syndrome is related to the consumption of sugar-sweetened beverages [46,47] and, in particular, a high intake of fructose-containing drinks and foods in the general population is associated with induction of lipogenesis with hypertriglyceridemia, and insulin resistance, paralleled by oxidative stress, which is a relevant factor contributing to the glycation process [48,49,50]. Indeed, plasma and tissue AGE accumulation has been reported in animal models of high fructose consumption [12,51], while experimental data on in vivo fructose-derived glycation consequences are still limited.

## 3. Dietary Sugar-Induced Glycation: Interference on Cell Functions

In addition to the effects attributed to the excess of calorie intake induced by high sugars consumption on metabolism, the sugar-derived AGEs have been shown in animal models to contribute to the development of pathological metabolic conditions through the interference with several protein functions and the activation of pro-inflammatory signals [52,53], as illustrated above in Scheme 1.

### 3.1. Interference with the SCAP/SREBP Lipogenic Pathway

Several studies have demonstrated that excessive fructose consumption is associated with increased ectopic lipid deposition in liver and skeletal muscle both in experimental models and in humans [48,49,54,55], and that fructose is able to promote hepatic lipogenesis [56,57] by inducing the expression and activation of transcription factors including sterol regulatory element-binding protein-1c (SREBP-1c) and carbohydrate response element binding protein (ChREBP) [52,58,59]. A positive correlation between lipogenesis and plasma triglyceride has been described in patients with isocaloric feeding added with high fructose level [60].

Besides the alterations of plasma lipid profile, fructose is suggested to be an important risk factor for the development of NAFLD [11]. The pathogenic mechanisms related to intrahepatic fat content induced by fructose consumption are related to an imbalance among fatty acid synthesis, beta-oxidation and triglyceride outflow from the liver [24]. Indeed, the direct comparison between saturated fats- and fructose-rich diets revealed that these two dietary components differently affect liver lipid metabolism, with fructose enhancing both beta-oxidation and fatty acids export, counteracted by a strong activation of lipogenesis and palmitate production [53].

The fructose-induced ectopic lipid deposition has been attributed to the activation of the transcription factor SREBP1c, which regulates the expression of several enzymes responsible for fatty acids de novo synthesis [61]. In studies directly comparing the effect of pure fructose and glucose in lipid metabolism a greater impact of fructose on lipogenesis activation has been demonstrated [48]. In particular, the chronic consumption of fructose- and glucose-sweetened beverages in mice revealed activation of the SCAP/SREBP pathway was, to a greater extent, in fructose-drinking mice [12]. The lipogenesis activation was associated with a different pattern of AGEs in the plasma and liver of sugar-drinking mice, with a higher amount of glyoxal derived AGEs, such as glyoxal-lysine dimer (GOLD) and carboxymethyl lysine (CML), which are more resistant to AGE-degrading enzymes, in the livers of the fructose group. The hypothesis that excessive intake of dietary sugars might interfere with lipid metabolism through the action of AGEs has been confirmed by the observation that the SREBP-activating protein SCAP is highly glycated by CML, a modification evoking prolonged activation of SCAP by inhibiting its degradation [12,52,62].

The same mechanism has been proposed to enhance intramyocellular lipid deposition in the skeletal muscle of high-fructose consuming mice [52]. Interestingly, in skeletal muscle of mice, the overactivation of lipogenesis induced by high fructose intake was accompanied by modifications of muscle metabolic reprogramming and mitochondrial functions that were effectively reverted by the administration of an anti-glycative agent, demonstrating the relevant impact of fructose-derived AGEs on tissue specific signaling pathways.

### 3.2. Interference on Sphingolipids Metabolism

The deregulated enhancement of the de novo lipid synthesis can have relevant repercussions on the overall lipids metabolism. Recently, alterations of the sphingolipid metabolism have been evidenced in obese and diabetic patients, with increased plasma levels of ceramide and sphingosine-1-phosphate, which are hypothesized to elicit an inflammatory condition [63,64]. In animal models of the western diet, high in fats and fructose, increased ceramide de novo synthesis has been reported, in relation with reduced insulin sensitivity [65,66]. In parallel, two very recent studies highlighted a possible role for diet-derived AGEs on this unbalance among sphingolipid intermediates through the action of the AGE-receptor RAGE [67,68]. In particular, according to the work from Geoffrey and colleagues, the dose- and time-dependent effect of exposition to AGEs on mesangial cells proliferation was mediated by the modulation of the sphingolipids intermediates ceramide/sphingosine/sphingosine-1-phospahate and activities of related enzymes [67]. Similarly, the findings from Chen et al. demonstrated that polydatin, through its anti-glicative effect, evokes the reduction of sphingosine kinase activity and its byproduct sphingosine-1-phosphate, and this may be the underlying mechanism for the prevention of diabetic nephropathy and glomerular mesangial cells’ pro-fibrogenic signalling [68]. The pro-inflammatory profile induced by high levels of ceramide and sphingosine-1-phosphate has been demonstrated to contribute to cardiac impairment and peripheral insulin resistance, and the modulation of enzymes involved in their accumulation has been shown to ameliorate metabolic derangements [69,70]. However, the beneficial effects of prevention of AGE accumulation induced by high fructose intake on sphingolipids metabolism remains to be explored.

### 3.3. Interference on Inflammatory Response

Since the last few years, there has been strong evidence for a causative association between central (visceral) obesity and the development of type 2 diabetes and cardiovascular complications, though the mechanisms are not fully understood. However, in this context, a low-grade, chronic inflammation orchestrated by metabolic cells in response to excess nutrients and energy in metabolic tissues including adipose, liver, muscle, pancreas, and brain, has been defined as a causative factor underlying metabolic dysfunctions development and related comorbidities [71].

Besides indications that a high fructose intake is also associated with the onset of a generic inflammatory response in several tissues, recent studies have addressed the specific activation of the NLRP3 inflammasome complex in models of a high-fructose diet [25,53,72]. In a comparative study performed on mice fed a normal or a western-style diet, associated or not to fructose-sweetened beverages, the peculiar effect of fructose drinking was the renal activation of NLRP3 inflammasome [73]. Indeed, the targeting of inflammasome activation by antioxidant and antidiabetic compounds or selective NLRP3 inhibitors, has been demonstrated to be effective in reducing the inflammatory response in kidneys, hearts, livers, skeletal muscles, and brains of mice fed a fructose-containing diet [74,75,76,77,78]. The inflammasomes are multiporotein platforms activated by interaction of a variety of danger signals with membrane and cytoplasmic receptors. Among these receptors, a role for two AGE receptors, namely RAGE and Galectin-3, in inflammasome activation has been proposed [79,80,81,82]. However, there is still contrasting data about the exact mechanisms by which both Galectin-3 and RAGE act on inflammasome assembling. If the pro-inflammatory effect of RAGE signalling through activation of the NFkB pathway is already well known [83], whether Galectin-3 exerts a positive or negative stimulus for NLRP3 activation is still under debate [84,85]. In this perspective, further investigations are needed to definitely clarify whether the fructose-induced inflammasome activation is mediated by AGE accumulation and to understand the involved mechanisms.

### 3.4. Interference with Mitochondrial Function and Oxidative Stress

Mitochondrial dysfunction and oxidative stress are strictly interconnected events representing the common features of metabolic disorders and chronic inflammatory diseases. It has been reported that a high intake of dietary sugars can evoke a mitochondrial overload in tissues with a high rate of energy metabolism, such as liver and cardiac and skeletal muscle, leading to enhanced mitochondrial respiratory chain activity and oxidative stress [65,86,87]. In particular, complexes I and III being the key-point for reactive oxygen species production, the enhancement of their activity due to increased energy influx results in oxidative stress, which, in turn, can compromise the activity of the iron-sulfur center enzymes, such as the complex I and II, and the complex III itself [88]. It is well known that oxidative species favour the glycoxidation reaction of proteins in the presence of reducing sugars, thus resulting in AGE accumulation. In turn, AGEs exert a pro-oxidant effect compromising antioxidant enzymes activity and mitochondrial functions, thus creating a vicious cycle [89]. In this regard, a very recent study evidenced that fructose feeding activates in rats the so-called AROS axis, featured by the consecutive enhancement in plasma AGEs level—tissue RAGE activation—mitochondrial ROS production, with subsequent intracellular AGE formation [44]. In particular, recent research has provided evidence that overconsumption of carbohydrates in the diet, especially sugars, may represent a risk factor for neurodegenerative diseases through the development of mitochondrial dysfunction, oxidative stress, and inflammatory reaction, and the cerebral accumulation of AGEs is considered a key mediator [90,91,92].

In addition, further recent findings highlighted in models of high-fructose intake the impairment of the transcriptional activity of the nuclear factor erythroid 2-related factor 2 (Nrf2), a central player in the regulation of many antioxidant enzymes, including glyoxalase-1, the main enzyme responsible for AGE detoxification [53,92,93], suggesting a twofold contribution of fructose on glycation through both enhanced production and reduced detoxification of AGEs.

## 4. Dietary Sugar-Induced Glycation and Pathogenic Role in Diseases

### 4.1. Data from Animal Studies

As mentioned above, the glycation induced by dietary sugars, through interference with many cell functions and signalling pathways, may contribute to the development of tissue damage and organ disease. However, most research studies on the pathogenic role of endogenous sugar-derived glycation have been performed in animal models, often through the employment of very high doses of sugars, particularly of fructose, not comparable to the general human nutrition.

Notably, in animals, the metabolic outcomes of a caloric restriction were attenuated by the diet enrichment with methyl glyoxal, demonstrating that dietary AGEs can induce oxidative stress, insulin resistance, and profibrotic conditions independently from total calorie amount [94]. In addition, in animal models, an involvement of AGEs specifically derived from dietary sugars in metabolic disturbances or in organ dysfunction has been demonstrated by the use of anti-glycative agents. Betanin, an antioxidant compound also provided with anti-glycative properties in vitro, prevented in rats’ hearts the collagen cross-link and expression of markers of fibrosis that were increased after 60 days of drinking 30% fructose solution [95]. In a very recent work, the administration to rats of the PPARγ agonist Rosiglitazone was able to improve most of the signs of metabolic syndrome induced by a 60% fructose drinking for 21 days, through the reduction of urine and plasma AGE levels and of kidney and liver RAGE expression [44]. Our research group has previously reported the beneficial effects of a specific anti-glycative compound, pyridoxamine, in a mouse model of metabolic syndrome induced by a 12-week 60% fructose diet, where the prevention of AGE accumulation in plasma, skeletal muscle, and brain was paralleled by an improvement of systemic glucose and lipid metabolism and reduction of inflammatory and oxidative stress markers and restoration of mitochondrial function in skeletal muscle and brain [52,92]. Pyridoxamine administration has also been found to be effective in the amelioration of glucose homeostasis and insulin sensitivity, and in the prevention of visceral adipose tissue expansion and local expression of inflammatory markers in a mouse model of high-fat diet, suggesting an involvement of diet-induced endogenous formation of AGEs in the pathogenesis of obesity [96]. In this regard, in mice with genetically-induced deletion of leptin receptors Db^-/-^), which are prone to consume excessive calories and develop obesity and insulin resistance, high levels of CML were trapped by the adipose tissue, while the deletion of RAGE reverted CML accumulation in adipose tissue, increasing its plasma levels, indicating a RAGE-dependent mechanism underlying endogenous AGE-induced obesity [97].

### 4.2. Data from Human Studies

On the other hand, epidemiological and observational studies in humans have not univocally demonstrated a relation between high sugar intake and organ diseases, such as liver steatosis, cardiac impairment, or neurodegeneration, so far. Excessive soft drink consumption has only been associated with increased risk for metabolic disorders such as obesity and insulin resistance [98,99], but their effects are often attributed to an unspecific calorie excess [100,101]. In this regard, two recent studies revealed that an isocaloric fructose restriction, where the calories of fructose were substituted by starch, was sufficient to improve metabolic parameters and lipoprotein markers of CVD risk, in particular reducing apoC-III, which has been associated with atherogenic hypertriglyceridemia, in children with obesity and metabolic syndrome, irrespective of weight change, indicating that the detrimental effects of sugar, specifically fructose, are independent of its caloric value or effects on adiposity [102,103]. Moreover, a very recent double-blind randomized crossover trial analysed acute metabolic and endocrine responses induced by fructose and glucose load in healthy young subjects and showed that fructose leads to unfavorable modifications of some metabolic parameters, including increased serum concentrations and 3 h-AUC of uric acid, aldose reductase, and lactic dehydrogenase, increased systolic blood pressure, and decreased endothelial nitric oxide production in comparison with the same amount of glucose [104].

Nevertheless, the involvement of endogenous glycation in dietary sugar-induced dysmetabolism is far from being demonstrated in humans. Though, the reduction of dietary (exogenous) AGEs in type 2 diabetes or obese patients, without modification of the total calories intake, has also been demonstrated to be effective in amelioration of insulin sensitivity, with reduction of inflammatory markers and restoration of AGEs detoxifying systems and mitochondrial metabolism regulators [42,105]. In this regard, to induce the expression and activity of the AGE-detoxyfying enzyme glyoxalase-1, through the synergic action of trans-resveratrol and hesperetin, is likely to be a promising strategy to prevent the onset of insulin resistance and vascular inflammation in overweight individuals [106].

Moreover, endogenous and exogenous AGEs have been related to cognitive decline and impaired memory in two different studies, indicating that serum levels of methylglyoxal in elderly individuals were directly correlated with dietary AGE intake and cognitive decline assessed by the Mini Mental State Examination [107,108].

Recent cross-sectional studies from DeChristopher and colleagues indicated that the consumption of HFCS or fructose-sweetened beverages is associated with asthma and bronchitis in adults and to asthma in children [109,110,111]. Authors suggest an interesting mechanistic hypothesis, according to the in vitro observations of Bains and Gugliucci [112], of an intestinal formation of AGEs from excess free fructose intake, which may be absorbed in the circulation and induce a systemic inflammatory condition through the binding to RAGE, thus contributing to lung diseases and impaired immune response. This fascinating theory, described in detail in the very recent review from Gugliucci [113], however, is still not supported by in vivo experimental data.

## 5. Conclusions

In the present review, we provide experimental data and epidemiological observations indicating the negative effects of excessive intake of sugar-added foods and beverages, particularly of fructose. The data here reported also suggest that the glycation process following high sugar intake may play a central role in the development of metabolic disturbances by interfering with many cell signaling pathways and influencing the pro-inflammatory and pro-oxidant status that contributes to tissue injury and organ dysfunction.

However, these mechanisms need to be deepened through animal models trying to mimic the real human sugar consumption and aimed to clarify the significance of dietary sugar-derived AGEs in metabolic diseases and increase the transferability to the human nutrition.

Nevertheless, it is difficult to definitely establish limits for sugar intake above which risks for human health are increased. The guidelines to limit sugar addition in foods are not always free of conflicts of interest between public health and the food industry. Thus, since to date it is not possible to discriminate in human nutrition the contribution of different monosaccharides and the general calorie excess to protein glycation, high quality clinical trials are needed to evaluate the sugar daily intake, particularly of fructose, that can represent a feasible risk for human health, through the design of appropriated dietary interventions.
